# The anatomical pathology of gout: a systematic literature review

**DOI:** 10.1186/s12891-019-2519-y

**Published:** 2019-04-01

**Authors:** Patapong Towiwat, Ashika Chhana, Nicola Dalbeth

**Affiliations:** 1 Department of Medicine, University of Auckland, Auckland, New Zealand; 2 Department of Medicine, Naresuan University, Phitsanulok, 65000 Thailand

**Keywords:** Gout, Pathology, Histology, Tophus, Synovium

## Abstract

**Background:**

The aim of this systematic literature review was to comprehensively describe the anatomical pathology of tissues affected by gout.

**Methods:**

We searched PubMed, The Cochrane Library, Excerpta Medica Database (EMBASE), and Web of Science Core Collection for all English language articles published before March 2018. Articles were included if they described the microscopic or macroscopic appearances of gout in human tissue.

**Results:**

Four hundred and seventeen articles met inclusion criteria and were included in the review. Articles describing the anatomical pathology of gout in musculoskeletal structures, including bone, tendon and ligaments, synovium and cartilage, were most common. Articles describing skin and kidney pathology in gout were also common, with pathology in other sites such as visceral organs less common. At all sites, monosodium urate crystal deposition was reported, and the tophus was also described within many different tissues. During a gout flare, diffuse acute neutrophilic synovial inflammation was evident. The tophus was described as an organised chronic giant cell granulomatous structure consisting of monosodium urate crystals, innate and adaptive immune cells, and fibrovascular tissue.

**Conclusions:**

Consistent with the clinical presentation of gout, most studies describing the anatomical pathology of gout report involvement of musculoskeletal structures, with monosodium urate crystal deposition and tophus the most common lesions described. This review details the anatomical pathology features of gout at affected sites.

**Electronic supplementary material:**

The online version of this article (10.1186/s12891-019-2519-y) contains supplementary material, which is available to authorized users.

## Background

Gout is a common cause of inflammatory arthritis. Among the US adult population, the prevalence of gout is 3.9% [1]. Monosodium urate (MSU) crystal deposition is the central pathophysiological cause of the disease. Typically, the clinical course of gout includes asymptomatic hyperuricemia, intermittent attacks (flares) of acute arthritis, intercritical gout, and, if hyperuricaemia is untreated, advanced gout, characterized clinically by tophi, chronic gouty arthritis and joint damage in some individuals [2].

Acute onset of intensely painful monoarthritis, usually affecting the lower limb and most often the first metatarsophalangeal joint, is the classical clinical presentation of gout [3]. The pain of the acute flare usually peaks within 24 h and gradually resolves over 7–14 days [4, 5]. In the setting of an acute inflammatory monoarthritis, concerns about other diagnoses such as septic arthritis may necessitate pathological examination of the affected tissue. Furthermore, although the presentation of gout is usually quite characteristic, patients may present with atypical symptoms such as subcutaneous nodules, prolonged joint inflammation, or acute inflammation at uncharacteristic sites [6]. While microscopy of aspirated material for crystal confirmation or advanced imaging methods may assist with the diagnosis, pathological analysis of affected tissue may be required to confirm the diagnosis. The aim of this systematic literature review was to describe comprehensively the anatomical pathology of gout, including the macroscopic appearances, light microscopy (including immunohistochemistry) and electron microscopy.

## Methods

Searches were performed in the following electronic databases: PubMed, Excerpta Medica Database (EMBASE), and Web of Science Core Collection. The following search keywords were used: “gout or gouty”, “pathology or pathological or pathologies or histology or histological or histologies”. An example of the full search strategy listed is shown in the Additional file 1. Articles were included if they described the microscopic or macroscopic tissue appearances of gout in humans. Articles were excluded if they were not published in the English language or reported cytological analysis only. Bibliographical references of individual publications were also checked. Data sources were English publications from these databases, and hand searches. No date restrictions were used; the earliest database search date was 1872. The search was undertaken in July 2016, with an updated search in March 2018 to ensure the analysis findings were up to date. Two authors (PT and ND) reviewed all articles. In the event of disagreement regarding inclusion criteria, the article was reviewed by both authors to gain consensus.

Information regarding the pathological features of gout was extracted from each article in a standardized form, along with information about the organ or tissue examined, fixative for microscopy, and specific features described for the following categories: macroscopic appearances, light microscopic appearances, immunohistochemistry, and electron microscopy. Information was then summarised for each pathological feature and for each tissue. In order to avoid redundancy of references, the first available description of each finding is cited in this review.

For pictorial representation of the key findings identified during the review process, representative images of joints affected by microscopically proven gout (from a first metatarsophalangeal joint, a finger proximal interphalangeal joint, a finger distal interphalangeal joint, and a knee) and a tophus sample were identified from two patients with gout undergoing orthopaedic surgery and two cadaveric donors with microscopically-proven gout. Human sample collection was approved by the Northern Regional ethics committee and all patients provided written informed consent. Collection and use of human cadaveric tissue was in accordance with the New Zealand Human Tissue Act 2008. Cadaveric samples were transferred to 70% ethanol immediately after collection and all samples were demineralised at room temperature in 10% formic acid and embedded in paraffin. Slides with 4 μm tissue sections were prepared and then stained with haematoxylin and eosin or toluidine blue, and examined using polarising light microscopy. Immunohistochemistry for tartrate resistant acid phosphatase (TRAP) was undertaken as previously described [7].

## Results

### Search results

**Fig. 1:**
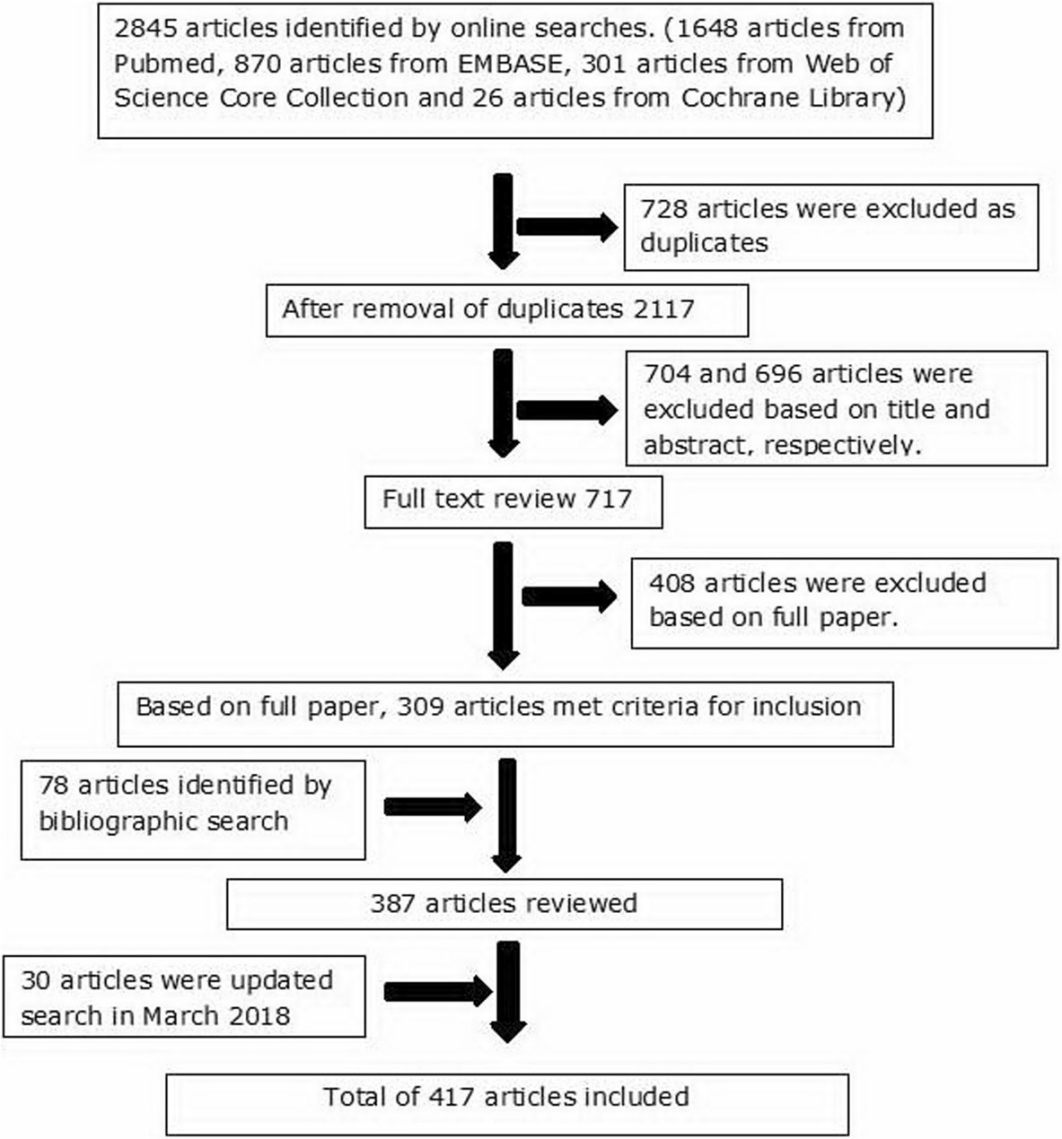
Summary of literature search results

A total of 2845 articles were identified by the search. Duplicates (728 articles) were excluded. A total of 1400 articles with titles and abstracts that did not relate to anatomical pathology features of gout were excluded. A further 408 articles were excluded after full text review. The literature review included total of 387 articles that were identified by databases (309 articles) and bibliographic searches (78 articles). A further 30 articles were identified in the updated search in March 2018. Therefore, the literature review included a total of 417 articles. The results of the searches are shown outlined in Fig. 1.

A summary of the extracted literature is shown in Tables 1 and 2. The most frequently involved tissues were synovium, bone, skin, cartilage, tendon, ligament and kidney, with less commonly affected musculoskeletal tissues shown in Table 1 and non-musculoskeletal tissues shown in Table 2. Methods of fixation were described in 133/417 (31.9%) articles, with 49 articles describing ethanol fixation, and 79 articles describing formalin fixation, and five articles describing other fixation methods. Microscopic confirmation of MSU crystals was reported in 402/417 (96.4%) articles.

**Table 1:** Summary of articles describing musculoskeletal tissues in gout

Tissue involvement	Macroscopic appearances	Light microscopic appearances	Immunohistochemistry study	Electron microscopy appearances
Synovium	16	34	3	3
Bone	83	103	2	0
Cartilage	12	10	1	0
Skin	22	78	0	0
Tendon and ligament	45	36	1	0
Joint capsule	1	3	0	0
Muscle	4	3	0	0
Bursa	4	2	0	0
Meniscus	1	1	0	0
Carpal tunnel	21	13	0	0
Spine	63	68	0	0

**Table 2:** Summary of articles describing involvement of non-musculoskeletal tissues or organs in gout

Tissue involvement	Macroscopic appearances	Light microscopic appearances	Immunohistochemistry study	Electron microscopy appearances
Eye	3	10	0	3
Nose	1	2	0	0
Paranasal area	1	1	0	0
Middle ear	1	3	0	0
Larynx	3	4	0	0
Nail	0	1	0	0
Nerve	2	1	0	0
Breast	2	5	0	0
Heart	9	10	0	0
Coronary artery	0	1	0	0
Lung	2	2	0	0
Small intestine	2	3	0	0
Colon	2	2	0	0
Mesentery	2	2	0	0
Pancreas	1	1	0	0
Peritoneum	1	1	0	0
Kidney	14	37	3	2
Prostate	0	2	0	0
Urinary bladder	1	0	0	0

Review of the extracted literature demonstrated that the features of MSU crystal deposition or tophus were described in many different tissues. Given the consistent appearance of these features at different sites, the anatomical pathological features of the MSU crystal deposition and tophus are described first and separately.

### MSU crystal deposition

Macroscopic appearances describing MSU crystal deposition were reported in 213 articles. Light microscopic appearances were reported in 325 articles. The electron microscopy appearances were described in two articles. Only 15 articles of the 417 (3.6%) articles did not report MSU crystal deposition.

#### Macroscopic appearances

**Fig. 2:**
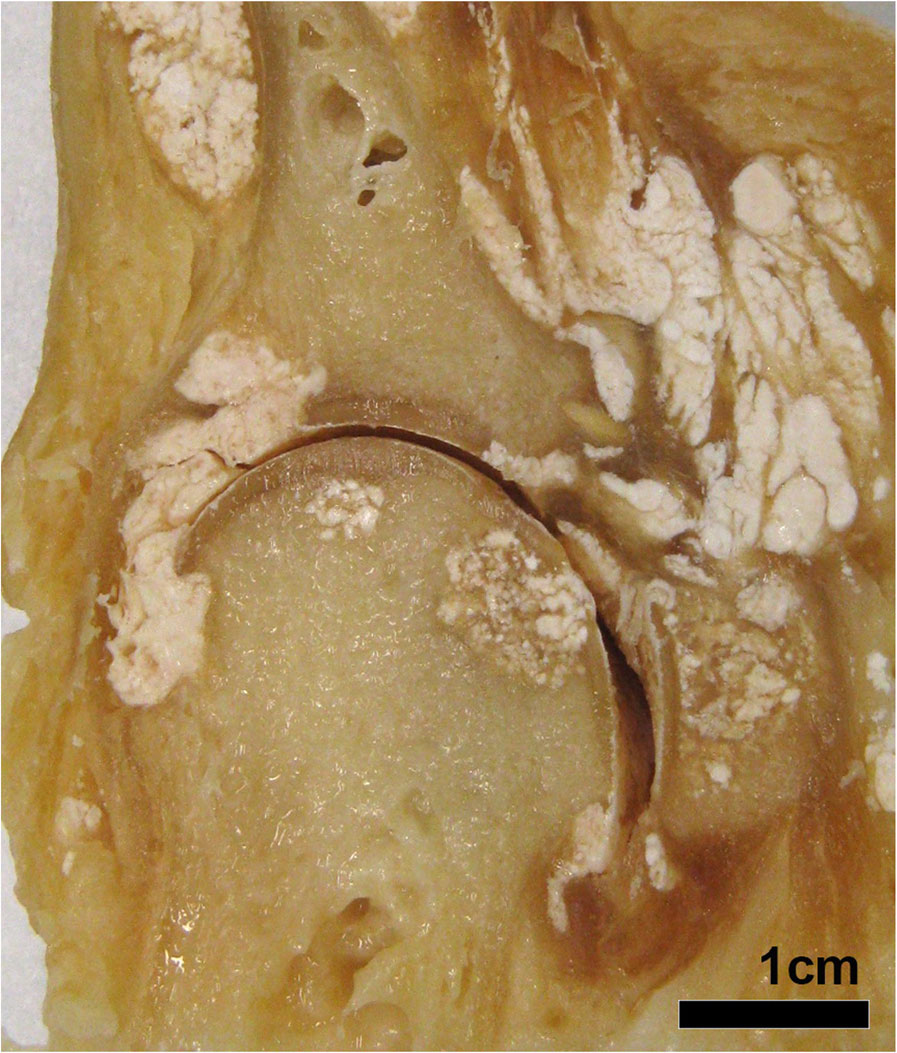
Photograph showing MSU crystal deposition, tophus and joint damage at the macroscopic level (sagittal plane) in the left first metatarsophalangeal joint from a cadaveric donor with tophaceous gout. Bone erosion and cartilage damage adjacent to MSU crystal deposition and tophus can be seen. Fibrous septae are also evident between deposits of MSU crystals within the tophus

MSU crystals were typically described as being white in colour [8] (Fig. 2). The appearances of sugar icing [9], chalky [8], snow-like [10], powdery [11], or toothpaste-like material [12] were also reported.

#### Light microscopic appearances

**Fig. 3:**
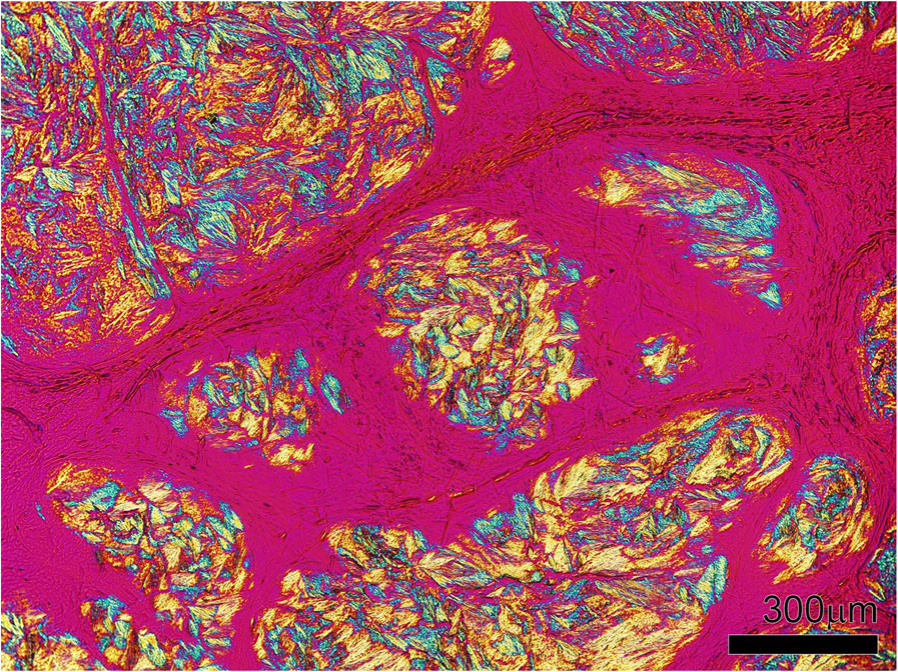
Collections of birefringent MSU crystals within an olecranon bursa tophus. Tissue section is unstained and viewed using polarizing light microscopy with a red compensator

Rod [8] or long needle shaped crystals [13] were reported. Average length was about 1/1500 of an inch (16.9 μm) [8]. Under light microscopy, collections of crystals had many variations of colour, including eosinophilic [13], basophilic [14], grayish [15], and colourless [16]. Under polarizing light, MSU crystals showed negative birefringence [17] (Fig. 3).

#### Electron microscopy appearances

On electron microscopy, hexagonal, octagonal, or cylindrical crystals were observed [18]. Long needle shaped rods were also described [19]. The length was measured as 1-5 μm [18]. However, lengths up to 80 μm were also reported [18]. In cross-section, MSU crystals had a regular lattice structure, formed in sheets that were 50 Å thick [18].

### Tophus

Macroscopic appearances describing the tophus were reported in 203 articles. Light microscopic appearances were reported in 304 articles. Immunohistochemistry study and electron microscopy appearances were described in 13 and five articles, respectively. Seven of the 417 (1.7%) articles did not report tophi.

#### Macroscopic appearances

The tophus was described as a chalky white nodule [8] (Fig. 2), with variable size and shape of the tophus [20]; irregular [21], nodular [8], plaque-like [22], cauliflower [23], fungiform [23], exophytic [24] and multilobular [25] lesions reported. The consistency also varied, ranging from firm [26], gritty [14] semi-solid [8], cheesy-like [27] to gelatinous [28]. MSU crystals were visible surrounded by a thin walls of fibrous tissue dividing the tophus into multiple compartments [29]. Some tophi were also encapsulated [29].

#### Light microscopic appearances

**Fig. 4:**
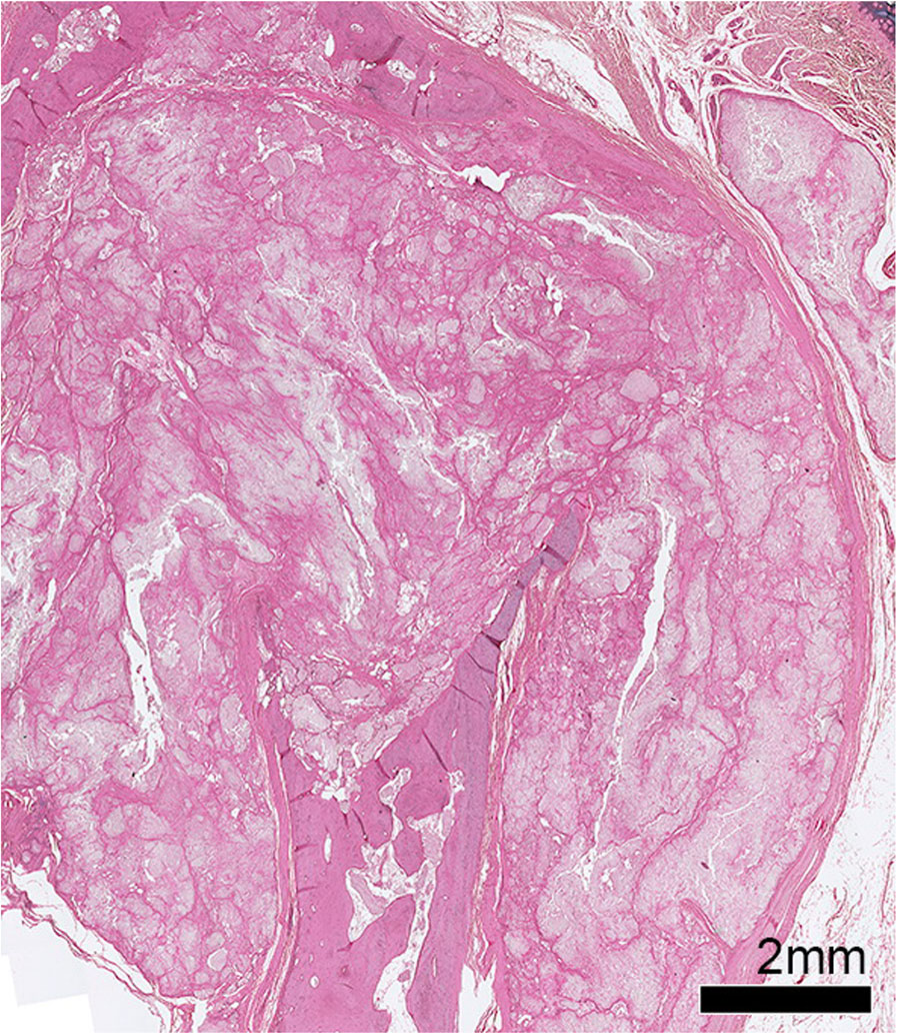
Tophus associated with structural damage in a right little finger distal interphalangeal joint from a cadaveric donor with microscopically proven gout. The joint architecture has been lost, with tophus and significant bone erosion present. Tissue is stained with haematoxylin and eosin and viewed by light microscopy

On light microscopy of the tophus, an organized structure was described [30] (Figs. 3 and 4). A crystalline centre was observed, as an acellular (‘necrotic’) collection of MSU crystals [29] (Fig. 3). The crystalline centre was surrounded by a corona zone consisting of multinucleated giant cells of the foreign body type, histiocytes, fibroblasts, lymphocytes and plasma cells [13, 31, 32]. The corona zone was surrounded by a fibrovascular zone that was sparsely infiltrated with mononucleated cells, representing the fibrous septae that are visualized macroscopically. Calcification was also observed in some tophi [33].

#### Immunohistochemistry

Within the central crystalline core of the tophus, silver nitrate staining was observed, but Congo red, Alcian blue, von Kossa stain, and Periodic acid-Schiff (PAS) staining were negative [34]. IgM and IgG staining was also present in the crystalline area [35]. Less intense staining for IgA was described in the same locations. These immunoglobulins were distributed homogeneously [35]. Positive MAb UCHMI was reported in the central zone, consistent with monocyte-derived debris in this area [30].

Many corona cells stained positively for CD68, indicating cells of the monocyte/macrophage lineage [36]. Macrophages in the corona zone expressed the CR3 complement receptor [37]. In the corona zone, CD4+ T cells [38], CD8+ T cells [39], CD20+ B cells [39] and mast cells [39] were also observed. Neutrophils were rarely observed. Staining for pro-inflammatory factors including interleukin (IL)-1β [39], IL-6 [38], S100A8 [36], S100A9 [36] and tumor necrosis factor-α (TNF-α) [38] was observed in cells within the corona zone. Transforming growth factor-1 (TGF-1) expressing mononucleated cells were also observed in the corona zone [39]. Multinucleated cells within the corona zone expressed the osteoclast phenotype markers including cathepsin K, the αv component of the vitronectin receptor (CD51), tartrate-resistant acid phosphatase (TRAP) [7], and receptor activator of nuclear factor ΚΒ (RANK) [38]. Strong staining for matrix metalloproteinase (MMP)-9 and weak to moderate staining for MMP-2 was described in the corona zone macrophages [36].

Cells within the fibrovascular zone were positive for MAbs2D1 (CD45, leucocyte-common antigen), UCHM1 (monocytes principally) and 52 (HLA-D antigens) [37]. The CD68+ cells in the fibrovascular zone were mononucleated [39]. Mast cells were also identified in fibrovascular zones. Neutrophils were rarely observed [39]. However, T cells including CD3+ and CD8+, plasma cells and CD20+ B cells were also seen in the fibrovascular zone [39]. In the fibrovascular compartment, a moderate number of cells with positive immunoreaction for TNF-α, MMP-2, MMP-9, bcl2 and bax were demonstrated [36]. IL-1β and TGFβ1-expressing cells were identified at lower densities than the corona zone [39]. Receptor activator of nuclear factor KB ligand (RANKL) was strongly expressed in T cells [38]. In contrast, there was minimal osteoprotegerin (OPG) staining [38].

#### Electron microscopy appearances

In the tophus, crystals were observed in various arrangements, including parallel to each other, interspersed with mature collagen fibers and thinner fibrils without periodicity, or randomly [40]. All crystals were in an amorphous matrix [40]. Cells adjacent to the crystals had prominent rough endoplasmic reticulum and large lipid deposits [40]. These cells contained lucent patches of cytoplasm suggesting cell degeneration [41]. Occasional crystals were seen in small phagosomes of the cells [40].

### Synovium

Macroscopic appearances describing the synovium were reported in 16 articles. Light microscopic appearances were reported in 34 articles. Immunohistochemistry study and electron microscopy appearances were described in three articles. (Table 1).

Features of both acute and chronic synovial inflammation were described. As these appearances differed substantially, they are reported separately.

#### Acute synovial inflammation

Macroscopic appearances were described in 3 articles. Light microscopic appearances were reported in 13 articles. Immunohistochemistry and electron microscopy appearances were reported in one and three articles, respectively (Table 1).

##### Macroscopic appearances

The macroscopic appearances of MSU crystal deposition were described as white chalky material in the synovium [42]. Moderate hyperemia was observed in the synovial membrane [43].

##### Light microscopy

The major appearance of acute synovial inflammation was diffuse and perivascular inflammatory cell infiltration [40]. Neutrophils were the dominant cell type [44], often with evidence of degranulation [45]. Numerous capillaries were seen in the synovial membrane [42]. Synovial villi were observed, with neutrophils, mononuclear leucocytes and fibrin infiltrating the villi [40]. There was oedema, columnar swelling and proliferation of synovial lining cells [44]. These cells were seen infiltrating small vessel walls but no fibrinoid necrosis was present [40]. Although neutrophils were evident superficial to the vessel, lymphocytes also surrounded venules [41]. In addition to neutrophils, lymphocytes, macrophages and lesser numbers of plasma cells were also observed [40]. In some patients presenting with an acute flare of arthritis, microtophi and foreign body type giant cells were observed in the synovium [40, 44, 46]. Although crystals were observed in synovial tophi using polarizing light microscopy, free crystals were not observed in the intact synovial lining or other synovial sites [40].

##### Immunohistochemistry

MMP-1 mRNA was present in the lining layer and endothelial cell MMP-1 expression was also reported [47]. Weak perivascular staining for cathepsin B mRNA was observed [47].

##### Electron microscopy

Surface fibrin-like material was seen [40]. In the synovial membrane, type B lining cells (resembling fibroblasts) with prominent lipid deposits predominated [40]. The less common type A lining cells (resembling macrophages) often had large vacuoles containing finely granular material and cell debris [40]. Microtubules and microfilaments were seen in synovial lining cells [40]. In the superficial synovium, extracellular debris from necrotic cells was observed [40].

Beneath the synovial lining layer, neutrophils, other inflammatory cells and extravasated red blood cells were seen [40]. These cells were also seen around small vessel walls. Phagocytosed cell fragments and intact polymorphonuclear leucocytes were observed within tissue macrophages. In small vessels, multilaminated basement membranes, occasional endothelial necrosis and luminal obliteration by platelets, erythrocytes, and neutrophils were observed [40]. Electron dense extracellular deposits between endothelium and pericytes were also observed in occasional patients. Neutrophils within the venule lumen showed features of degranulation and fragmentation. Microtubules and microfilaments were observed in venular endothelium pericytes, but not in neutrophils within the inflamed synovium [40].

Although membrane bound and non-membrane bound cytoplasmic lucent clefts (suggestive of crystals) were observed in the synovial cells, free crystals were not observed within the acutely inflamed synovium, except in synovial tophi [40, 41]. Within synovial tophi, MSU crystals were identified in both extracellular and intracellular sites [41]. They were surrounded by and phagocytosed within fibrocyte like cells [40]. Crystals and crystal spaces had little electron density and had dense margins [40].

#### Chronic synovial inflammation

Macroscopic appearances were reported in 14 articles. Light microscopic appearances were reported in 21 articles. Immunohistochemistry was described in two articles. The electron microscopy appearances were not described (Table 1).

##### Macroscopic appearances

MSU crystals were present as chalky or yellow-white flecks in the synovial membrane [48]. The synovium appeared nodular [49], proliferative [50] or thickened [51].

##### Light microscopy

**Fig. 5:**
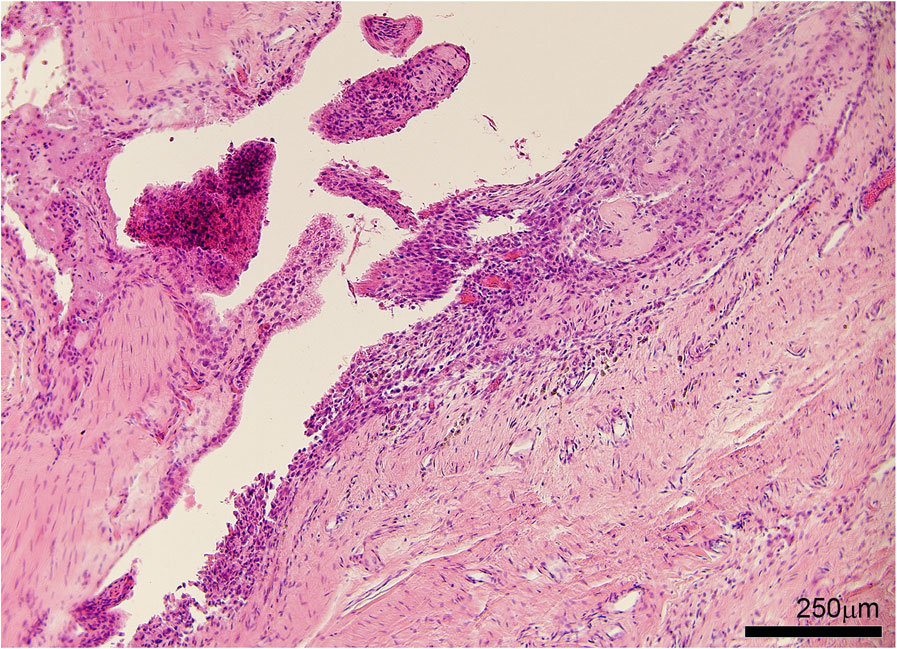
Chronic synovial inflammation with synovial microtophi in a right ring finger proximal interphalangeal joint from a cadaveric donor with microscopically proven gout. Tissue is stained with haematoxylin and eosin and viewed by light microscopy

Characteristic tophi were seen in synovium [16]. MSU crystals were also observed on the surface of chronically inflamed synovium [35]. The synovium appeared thickened [16] and proliferative, with pannus comprising vascular granulation tissue [52] or focal villous appearances [51] (Fig. 5). Some areas had thickened fibrosis [51] and loose stroma of cellular connective tissue [53]. Synovial lining cell proliferation, chronic synovitis with plasma cell infiltration [41], perivascular plasma cells [54], and a dense infiltrate of hemosiderin-laden macrophages [51] were also reported.

##### Immunohistochemistry

Crystals within the synovial tophus and on the synovial surface stained intensely for IgG and IgM, but less intensely for IgA. However, discrete immunoglobulin staining was not observed within inflamed synovium [35]. Diffuse CD3 staining was observed, but CD20 staining was rare [55].

### Bone

Macroscopic appearances were reported in 83 articles. Light microscopic appearances were reported in 103 articles. The immunohistochemistry findings were reported in two articles. The electron microscopy appearances were not described (Table 1).

#### Macroscopic appearances

Tophus within or adjacent to bone was observed [16] (Fig. 2). Irregular fragments of fibrous granulation tissue were seen [56]. Tophi were observed within bone erosions with loss of the normal structure of bone [32]. Bone destruction [48], necrosis [57], and pathological fracture [53] associated with tophi were also reported.

#### Light microscopy

MSU crystal deposition was seen in bone [32] and characteristic tophi were observed in the bone cortex and medulla [48] (Fig. 4). MSU crystal deposition was associated with cystic erosion [10]. Secondary cortical fracture [22] and focal destruction of bone trabeculae [58] associated with MSU crystal deposition were also described.

Some areas showed focal bone necrosis, with infiltration of trabecular bone by chronic inflammatory cells [59]. Although bones associated with tophus showed inflammation or multinucleated foreign body giant cells, other areas of subchondral bone and fatty marrow were relatively normal [16]. Fibrotic bone marrow with proliferation of dilated capillaries was described [11].

Osteoclastic bone destruction was observed [17]. At the bone-tophus interface, osteoclasts were present at the sites of bone erosion [60]. In bone adjacent to tophus, osteoblasts and lining cells were severely reduced compared with bone that unaffected by tophus [60]. Ankylosis [61] and new bone formation [17] with osteoblast rimming [62] were also described.

#### Immunohistochemistry

**Fig. 6:**
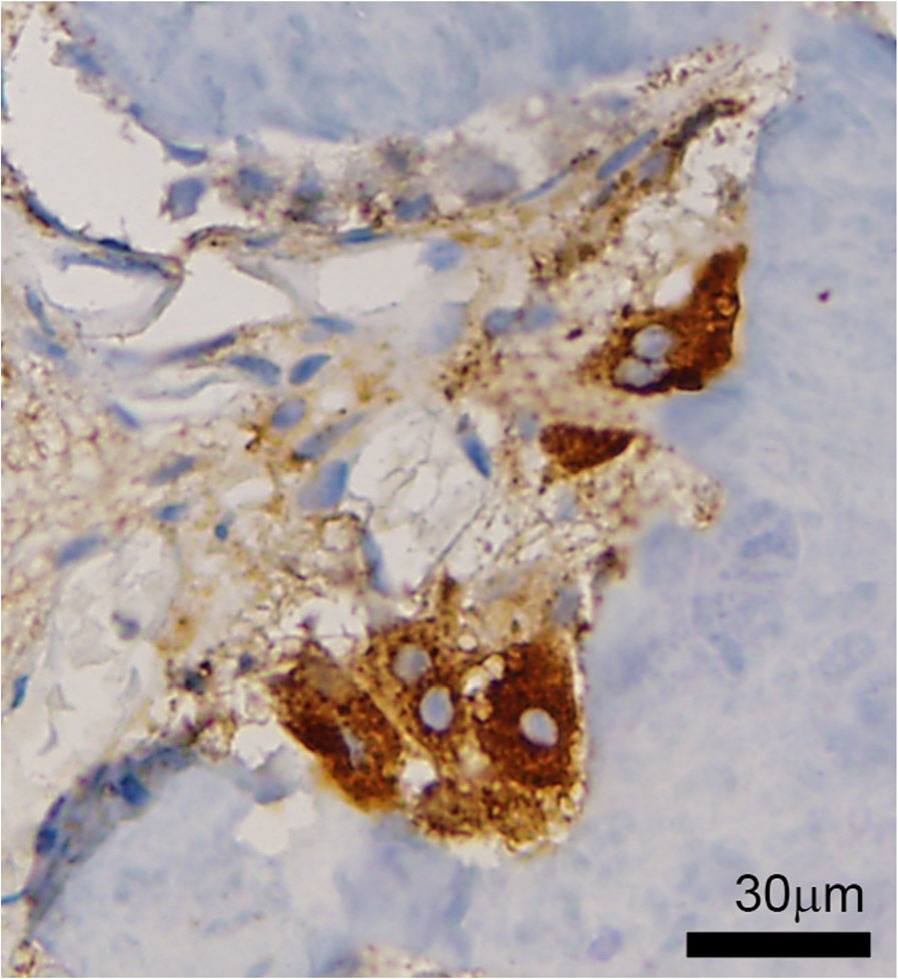
Immunohistochemistry analysis of a knee from a patient with gout stained with tartrate-resistant acid phosphatase (TRAP) demonstrating the presence of multi-nucleated osteoclasts (stained brown) at the bone–tophus interface

At the bone-tophus interface, multinucleated cells that expressed osteoclast phenotype markers including cathepsin K, TRAP, and CD51 were identified [7] (Fig. 6).

### Cartilage

Macroscopic appearances were reported in 12 articles. Light microscopic appearances were reported in 10 articles. There was one article that reported immunohistochemistry findings. The electron microscopy appearances were not described (Table 1).

#### Macroscopic appearances

Cartilage involvement was described as an early and common site of MSU crystal deposition in the joint [63]. MSU crystal deposition was visualized overlying cartilage, appearing as opaque, chalky white [32], or thin putty-like material [52]. Cartilage affected by MSU crystal deposition had a degenerative appearance [64]. Thinning [32], destruction [48] and partial erosion [52] of cartilage was also reported.

#### Light microscopy

**Fig. 7:**
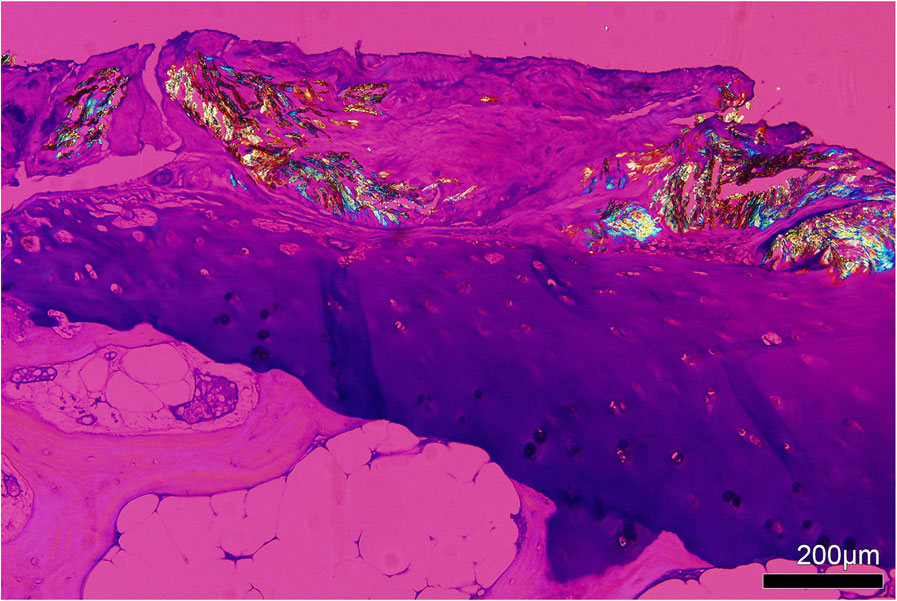
Toluidine blue stained tissue from left first metatarsophalangeal joint of a cadaveric donor with microscopically proven gout, showing MSU crystals deposited on the cartilage surface. Viewed using polarising light microscopy with a red compensator

Early light microscopy studies reported that MSU crystals within the joint were covered by a thin layer of cartilage, and that it was rare for MSU crystals to extend through the entire thickness of cartilage [63]. Extension of crystals into the lower zones of degenerative cartilage was described in subsequent reports [64]. Affected cartilage was described as fibrillated [16] fissured [64], eroded [65] or frayed [32], and replacement by fibrous tissue was observed [65] (Fig. 7). Granulation tissue or pannus was present near the articular edge and covering the surface of hyaline cartilage [32]. Empty chondrocyte lacunae and with few or no live chondrocytes were observed in cartilage fragments associated with tophi [66]. In cartilage fragments affected by gout, proteoglycan staining was preserved [66].

#### Immunohistochemistry

Type X collagen expression was present in affected cartilage [64]. S100 expression was observed throughout cartilage affected by gout, but at no different intensity to normal cartilage or control osteoarthritic tissue [64]. Compared with normal cartilage, cartilage affected by gout had high expression of superficial zone protein throughout all areas of cartilage, particularly in the superficial area [64].

### Tendon and ligament

Macroscopic appearances were reported in 45 articles. Light microscopic appearances were reported in 36 articles. There was one article that reported immunohistochemistry findings. The electron microscopy appearances were not described (Table 1).

#### Macroscopic appearances

Tophus deposition was observed both within [67] and around the tendon [68]. These tophi had a variety of appearances including creamy liquid [69], semi-firm [70] and gritty [71] texture. Involved tendons had a fusiform [72] or nodular [73] shape. Some tendons appeared hypertrophied [68] or thickened [74]. Tendon degeneration [73], with oedema, fragility, and loss of elastic character [75] was observed. Tendon rupture [76] and hematoma [68] were also reported.

#### Light microscopy

Tophi were described as both adjacent to and within tendon [48] and at the tendon-bone interface (enthesis) [77]. A focal acute inflammatory reaction was also described [69]. Affected tendons showed hyaline degenerative change [76], disorganised collagen fibrils [77] interstitial edema [78], fibrosis [75], and altered matrix mucopolysaccharide expression [78].

#### Immunohistochemistry

The inflammatory cell infiltrate within and surrounding tendons included CD68+ multinucleated cells and macrophages, with scatted lymphocytes including CD20+ cells within aggregates [77].

### Joint capsule

There was one article that reported macroscopic appearances. Light microscopic appearances were reported in three articles. The immunohistochemistry findings and electron microscopy appearances were not described (Table 1).

#### Macroscopic appearances

White ‘crystal-like’ material was observed in the joint capsule [79].

#### Light microscopy

Tophus consisting of MSU crystals surrounded by lymphocytes and macrophages was observed in the joint capsule [80].

### Muscle

Macroscopic appearances were reported in four articles. Light microscopic appearances were reported in three articles. The immunohistochemistry findings and electron microscopy appearances were not described (Table 1).

#### Macroscopic appearances

The macroscopic appearances of MSU crystal deposition were described within muscle. A white, discharging nodule in muscle was reported [69]. Hemorrhagic muscle was also described [81].

#### Light microscopy

Intramuscular crystal deposits were surrounded by macrophages and giant cells, with some superimposed acute inflammatory changes also observed at focal sites [69].

### Bursa

Macroscopic appearances were reported in four articles. Light microscopic appearances were reported in two articles. The immunohistochemistry findings and electron microscopy appearances were not described (Table 1).

#### Macroscopic appearances

In bursa, the macroscopic appearances of MSU crystal deposition [82] and tophi [83] were observed. The bursa appeared irritated and swollen [82].

#### Light microscopy

The microscopic appearances of tophus were described in olecranon and pre-patellar bursae [82].

### Meniscus

There was one article that reported macroscopic and light microscopic appearances. The immunohistochemistry findings and electron microscopy appearances were not described (Table 1).

#### Macroscopic appearances

The macroscopic appearances of MSU crystal deposition and tophi affecting the meniscus were reported [50].

#### Light microscopy

In the meniscus, MSU crystals were present on the surface of the meniscus, with the surrounding matrix appearing refractile and disrupted [84]. Matrix fibres appeared disordered with different sizes and orientations. Cells within the matrix were clustered and appeared hypertrophic. Aberrant vascular structures were also present [84].

### Spine

There were 63 and 68 articles that reported macroscopic and light microscopic appearances, respectively. The immunohistochemistry findings and electron microscopy appearances were not described (Table 1).

#### Macroscopic appearances

MSU crystals and tophi were seen in epidural space [85], epidural membrane [86], dura [87], ligamentum flavum [88], and intervertebral disc [48]. Tophus compressing the spinal cord structures including the cauda equina were described [88]. Tophi eroding bone were visualized causing pathological fracture [53].

#### Light microscopy

The microscopic appearances of tophi were observed in spinal structures including vertebral bone [89] epidural space [85], epidural membrane [86], dura [90], ligamentum flavum [88] and intervertebral disc [91].

### Carpal tunnel

There were 21 and 13 articles that reported macroscopic and light microscopic appearances, respectively. The immunohistochemistry findings and electron microscopy appearances were not described (Table 1).

#### Macroscopic appearances

The median nerve was compressed by tophi beneath the transverse carpal ligament [92] and associated with the flexor tendons within the carpal tunnel [72]. Crystal deposits were also viewed on the median nerve itself [93].

#### Light microscopy

The microscopic appearances of tophus were observed in structures within the carpal tunnel, including the flexor tendons beneath the median nerve [92] and the median nerve itself [93].

### Skin

There were 22 and 78 articles that reported macroscopic and light microscopic appearances, respectively. The immunohistochemistry findings and electron microscopy appearances were not described (Table 1).

#### Macroscopic appearances

Cutaneous tophi were described as thick walled [94], with gray-yellow, granular, firm and chalky appearance of fibrous tissue [48]. Ulcerated tophi were also described [32].

#### Light microscopy

The typical microscopic appearance of tophi were described within the skin [32], particularly within the dermis [95]. Areas of calcification [48], fat necrosis [96], granulomatous dermatitis [97], and hemosiderin deposition [98] were also reported. Inflammation was described in the squamous epithelium [99]. The affected skin had thin epidermis with loss of the normal characteristics of the epidermo-dermal junction [95], with hyperplasia, parakeratosis and hyperkeratosis also described [100]. In the superficial dermis, increased melanin pigment and pigment-laden melanophages were described [101]. The dermis had dilated blood vessels and swelling of endothelial cells [102]. There was a thickening of the wall of small and larger vessels in skin. These vessels were surrounded with various degrees of small round cell infiltrate [95].

### Kidney

Macroscopic appearances were reported in a total of 14 articles. Light microscopic appearances were reported in 37 articles. The immunohistochemistry findings and electron microscopy appearances were reported in three and two articles, respectively. A challenge to interpretation of the kidney pathology articles was the description of renal pathology in patients with gout and chronic kidney disease due to another cause. Reflecting the controversy about the existence of gouty nephropathy, it has been stated that “although mild interstitial renal disease does occur in a small proportion of patients with chronic gouty arthritis, the nephropathy generally results from hypertension, vascular disease, or independent renal disease [103].”

#### Macroscopic appearances

The size of involved kidneys varied from normal to atrophic [104]. The capsule had a rough, granular surface [105]. The cortex appeared irregular and congested, with both thinning and thickening observed [105]. Corticomedullary demarcation was lost in some patients [105]. The medulla appeared congested [32] and chalk white streaks were observed in the medulla [63], radiating to a peak at the papillae [105]. Round, smooth, white stones were seen in the tips of the pyramids [105]. Dilation and thickening of renal pelvis was described [106]. In a post-mortem case series of kidney pathology in patients with severe gout in the pre-urate lowering therapy era, kidney stones were observed in some patients, but were not universally observed [105].

#### Microscopic appearance

Features of nephrosclerosis were common [105]. Scarring and retraction of the cortex was common [105]. Glomerular swelling and thickening were described in some patients [105, 107]. Some glomeruli had hyaline change [53], vascular obliteration [105], atrophy and scarring [53]. Some parts of the cortex, especially the inner half, showed characteristic features of tophi including acidophilic material surrounded by foreign body giant cells and an infiltrate of mononuclear cells [32]. Irregular clusters of MSU crystals were also found in the cortex [32]. Atrophy and dilation of distal convoluted tubules were described [107]. Features of membranous nephropathy were also occasionally reported [108].

In the medulla, the deposition of “urates” with inflammatory reaction was found in various levels of medulla [105] and specifically within the tubules [106]. “Uric acid crystals” were also described in the renal tubules within the medulla [109] including the collecting tubules [104]. Characteristics of tophi were reported in the medulla [110]. Interstitial fibrosis and mononuclear cell infiltration were common [105]. The tubules showed fatty and hyaline degeneration [53], vacuolization [105], dilation [105] and distortion [111]. The tubules were filled with colloid casts, neutrophils and fragments of epithelial lining cells [105], Chronic pyelonephritis was also described [105].

Uric acid crystals combined with calcium crystals were observed in collecting tubules of some patients [104]. In the renal pelvis, submucosal fibrosis was present [106] and uric acid crystals were also seen [112].

In renal blood vessels, the intima of small and medium artery and arterioles was thickened by fibrous tissue [32]. Some vessels including arterioles and small arteries showed hyaline degeneration and occlusion [105]. Variable medial hypertrophy of arterioles was reported [113]. In the interlobular arteries, medial thickening [53], duplication of internal elastic lamina [53] and subintimal collagen [113] were described.

#### Immunohistochemistry

Reports of immunofluorescence have been in patients with gout and co-existent kidney disease, including cellular rejection and transplant glomerulopathy following renal transplantation [114], or drug-induced glomerulonephritis [115]. In patients with membranous nephropathy and gout, granular deposits of IgG, C3 and renal tubular epithelium antigen were observed in the glomerular capillary wall [108]. It appears that these findings are related to the primary kidney disease rather than gout, and the recent American Journal of Kidney Diseases Atlas of Renal Pathology has stated that immunofluorescence microscopy does not contribute to the diagnosis of gouty nephropathy [116].

#### Electron microscopy

In an electron microscopy analysis of renal biopsies from 13 patients with primary gout (all with serum urate above 8 mg/dL and 12/13 with tophi), and 11 people with essential hypertension but no gout, the electron microscopy appearances were similar between groups, with the exception of electron-opaque interstitial deposits of variable size in some of the gout cases [117]. Ultrastructural appearances of the glomerulus were often normal, and in areas that appeared normal on light microscopy, the tubules also appeared normal by electron microscopy. Increased mesangial matrix and thickening of the lamina densa was observed in both groups, and no other features were specific for gout [117]. In subsequent case reports of patients with primary gout, electron microscopy changes have been described, including diffuse cytoplasmic dense bodies and focal fusion of foot processes in the visceral epithelial cells of the glomerulus [107].

### Other organs

Macroscopic appearances involving other organs including in the head and neck, respiratory, cardiovascular and gastrointestinal systems were reported in a total of 33 articles (Table 2). Light microscopic appearances were reported in 51 articles. Electron microscopy appearances were reported in three articles. The immunohistochemistry findings were not described.

#### Macroscopic appearances

The characteristic macroscopic features of MSU crystal deposition and tophus were described in case reports of gout affecting other organs including the aortic [8] and pulmonary values [118], breast [119], lobes of the lungs [120], pancreas [121] and small intestine [120].

#### Light microscopy

Tophi were reported in cardiac structures including the mitral valve [13], aortic valve [122], pulmonary valve [118], and coronary artery [14]. Deposits of MSU crystals were also described in the prostate, surrounded by foreign body giant cells [48]. Deposits of MSU crystals were also described in the small intestine mucosa, submucosa, serosa and mesentery, with some fibrosis and chronic giant cell granulomatous inflammation [52]. Ocular involvement was also described with MSU crystals affecting the cornea [18], and tophi observed in the conjunctiva [123].

#### Electron microscopy

In the cornea, MSU crystals were either hexagonal, octagonal, or cylindrical in shape [18]. Occasionally, in conjunctiva, phagocytosed crystals within macrophages or extracellular crystal deposits were visualized by transmission electron microscopy [123].

## Discussion

In keeping with the clinical presentation of gout, most studies describing the anatomical pathology of gout report involvement of musculoskeletal structures, with other sites reported less frequently. MSU crystal deposition was almost universally described in affected tissues, consistent with the central pathogenic role of these crystals in gout. Although the pathological appearances of gout differed depending on the affected tissue, there were characteristic descriptions of the tophus at various sites, as an organised chronic giant cell granulomatous structure consisting of MSU crystals, innate and adaptive immune cells, and fibrovascular tissue.

Although MSU crystals are frequently observed in synovial fluid in the joint space and within synovial tophi, it is noteworthy that studies of synovial pathology during the gout flare did not demonstrate free MSU crystals within acutely inflamed synovium [40, 41]. Agudelo and Schumacher [40] postulated that a gout flare may be initiated by free MSU crystals interacting with synovial lining cells, and that following phagocytosis of MSU crystals, these lining cells may be rapidly released into the joint space.

Historical articles describing the anatomical features of disease can be aligned to contemporary advanced imaging studies of gout. An interesting observation in the studies of synovial pathology of gout was the reports of both acute neutrophilic synovitis, and chronic synovitis with microtophi and foreign body type giant cells. Recent advanced imaging studies have reported that many patients with gout have imaging evidence of synovitis during the intercritical period [124, 125], and that urate-lowering therapy can reduce MRI synovitis [125]. The description of the ‘double-contour sign’ on ultrasonography [126, 127] aligns with longstanding pathological observations that MSU crystals can be observed overlying hyaline cartilage. The close association between tophi and sites of bone erosion observed in dual energy CT studies [128, 129] has also been described in anatomical pathology studies, which have also demonstrated disordered osteoclast and osteoblast appearances at the sites of the tophus-bone interface.

The limitations of this study should be acknowledged. Since only English-language articles were included, some important studies were not included. Furthermore, although our search extended back to 1872, earlier descriptions of the pathological features of gout were not included in the analysis. Differences in sample preparation and changes in histological processing technologies over the review period may have led to some variation in pathological descriptions.

## Conclusions

In summary, this is the first systematic review of anatomical pathology in gout, describing, in detail, the macroscopic, light microscopic (with immunohistochemistry) and electron microscopic appearances of disease. This analysis emphasizes the central role of MSU crystal deposition as the pathogenic lesion in gout, the typical patterns of involvement on anatomical pathology assessment that mirror the clinical presentation of disease, and the characteristic tissue response to deposited crystals.

## Additional file

Additional file 1: Example search strategy from PubMed using Advanced Search. (DOCX 15 kb)

## Abbreviations

EMBASE: Excerpta Medica Database
IL: Interleukin
MMP: Matrix metalloproteinase
MSU: Monosodium urate
OPG: Osteoprotegerin
PAS: Periodic acid-Schiff
RANK: Receptor activator of nuclear factor KB
RANKL: Receptor activator of nuclear factor KB ligand
TGF-1: Transforming growth factor-1
TNF-α: Tumor necrosis factor- α
TRAP: Tartrate resistant acid phosphatase
