# Supplementary material for: The anatomical pathology of gout: a systematic literature review
Source: BMC Musculoskelet Disord. 2019 Apr 1;20:140. doi: 10.1186/s12891-019-2519-y (PMC6444644; doi:10.1186/s12891-019-2519-y)
Supplement: Supplementary file 1 — Example search strategy from PubMed using Advanced Search. (DOCX 15 kb) [file 12891_2019_2519_MOESM1_ESM.docx]

**Additional files**

**Supplementary material**

Example search strategy from PubMed using Advanced Search. The main keywords were searched in “ALL Fields” and mapped to the relevant ”MeSH terms” and “subheadings”.

| "gout"[MeSH Terms] OR "gout"[All Fields]) OR gouty[All Fields])  AND  "pathology"[Subheading] OR "pathology"[All Fields] OR "pathology"[MeSH Terms]) OR "pathology"[All Fields] OR "pathological"[All Fields]) OR "pathologies"[All Fields]) OR ("anatomy and histology"[Subheading] OR ("anatomy"[All Fields] AND "histology"[All Fields]) OR "anatomy and histology"[All Fields] OR "histology"[All Fields] OR "histology"[MeSH Terms]) OR histological[All Fields] OR ("histology"[MeSH Terms] OR "histology"[All Fields] OR "histologies"[All Fields]))  AND  ("humans"[MeSH Terms] AND English[lang]) |
| --- |
